# Molecular Role of Protein Phosphatases in Alzheimer’s and Other Neurodegenerative Diseases

**DOI:** 10.3390/biomedicines12051097

**Published:** 2024-05-15

**Authors:** Mubashir Hassan, Muhammad Yasir, Saba Shahzadi, Wanjoo Chun, Andrzej Kloczkowski

**Affiliations:** 1 The Steve and Cindy Rasmussen Institute for Genomic Medicine, Nationwide Children’s Hospital, Columbus, OH 43205, USA; saba.shahzadi@nationwidechildrens.org; 2 Department of Pharmacology, Kangwon National University School of Medicine, Chuncheon 24341, Republic of Korea; yasir.khokhar1999@gmail.com (M.Y.); wchun@kangwon.ac.kr (W.C.); 3 Department of Pediatrics, The Ohio State University, Columbus, OH 43205, USA; 4 Department of Biomedical Informatics, The Ohio State University, Columbus, OH 43210, USA

**Keywords:** protein phosphatase, amyloid beta, Alzheimer’s disease, neurodegenerative diseases, tau protein

## Abstract

Alzheimer’s disease (AD) is distinguished by the gradual loss of cognitive function, which is associated with neuronal loss and death. Accumulating evidence supports that protein phosphatases (PPs; PP1, PP2A, PP2B, PP4, PP5, PP6, and PP7) are directly linked with amyloid beta (Aβ) as well as the formation of the neurofibrillary tangles (NFTs) causing AD. Published data reported lower PP1 and PP2A activity in both gray and white matters in AD brains than in the controls, which clearly shows that dysfunctional phosphatases play a significant role in AD. Moreover, PP2A is also a major causing factor of AD through the deregulation of the tau protein. Here, we review recent advances on the role of protein phosphatases in the pathology of AD and other neurodegenerative diseases. A better understanding of this problem may lead to the development of phosphatase-targeted therapies for neurodegenerative disorders in the near future.

## 1. Introduction

Protein phosphatases (PPs) are enzymes that remove a phosphate group from the phosphorylated amino acid residues of substrate proteins [1]. Hundreds of biological targets are dephosphorylated by protein phosphatases as they create highly specialized holoenzymes with over 200 regulatory proteins [2]. The key reversible post-translational modification of protein phosphorylation and dephosphorylation regulates the shape, activity, localization, and stability of substrate proteins to regulate several regulatory circuits in eukaryotes [3]. While eukaryotic protein kinases appear to have evolved from a single progenitor [4], there are several potential sources from which protein phosphatases are thought to be recruited [5,6]. PPs exist in a variety of complexes with regulatory and targeting subunits that control the activity and specificity of catalytic subunits and target the enzymes to particular sites [7,8]. Phosphatases are classified into several categories, which are represented here, along with their therapeutic significance in human disorders. Protein phosphatases (PPs) are the key dephosphorylation effectors and are classified into three major types based on sequence, structure, and catalytic activity. These are the phosphoprotein phosphatase (PPP) family, which includes PP1, PP2A, PP2B, PP4, PP5, PP6, and PP7, and the protein phosphatase Mg^2+^- or Mn^2+^-dependent (PPM) family, which includes PP2C [9] (Table 1).

Alzheimer’s disease (AD) is a brain neurodegenerative disorder that slowly destroys social life, particularly memory [10]. It is estimated that over six million USA individuals with aged 65 years and older are affected by AD [11]. Furthermore, many people experience Alzheimer’s in their lives as family members and friends of those with this disease. The general symptoms of AD are variations in thinking, memory and a behavior known as dementia [12,13,14]. This is why the term “dementia” is occasionally used to refer to Alzheimer’s. However, AD is the most prevalent cause of dementia in older persons, although other diseases and disorders can also contribute to it. AD is not a typical aging process. It is the outcome of sophisticated brain alterations that begin years before the symptoms show up and cause the death of neurons and their interactions [15]. In AD, the tau protein undergoes hyperphosphorylation, forming soluble phospho-tau molecules which aggregate into paired helical filaments (PHFs) and produce neurofibrillary tangles (NFTs) [16]. Moreover, amyloid peptides are made by cleaving the amyloid precursor protein (APP) using β-site APP cleaving enzyme 1 (BACE1) and γ-secretase and are then excreted and deposited in extracellular senile plaques [17]. Protein kinases (PKs) and protein phosphatases (PPs) regulate the tau phosphorylation and dephosphorylation processes, respectively. Tau hyperphosphorylation and AD pathogenesis are aided by a functional imbalance between PKs and PPs [18,19].

**Table 1:** Comparison data of PP1–7 and their correlation with neurodegenerative diseases.

Protein Name	Amino Acid		Coding Gene		Disease	Ref
PP1	330		*PPP1CA*		AD, Parkinson disease (PD), Huntington’s disease (HD), Schizophrenia	[20,21,22,23]
PP2A	Structural	595	Structural	*PPP2R1A*	AD, PD, Down syndrome, Frontotemporal dementia, Glioblastoma multiforme	[24,25,26,27,28,29,30]
	Catalytic	309–310	Catalytic	*PPP2CA*		
	Regulatory	285–595	Regulatory	*PPP2R2A* *PPP2R2B* *PPP2R2C* *PPP2R3A* *PPP2R3B* *PPP2R5A* *PPP2R5B* *PPP2R5C* *PPP2R5D*		
PP2B	526		*PPP3CA*		AD, PD, HD Schizophrenia	[31,32,33]
PP4	Catalytic	320	Catalytic	*PPP4C*	AD	[34]
	Regulatory	1700, 500	Regulatory	*PPP4R2* *PPP4R3*		
PP5	525		*PPP5C*		AD, PD, HD	[35,36]
PP6	Catalytic	348	Catalytic	*PPP6C*	AD, PD	[34,37]
	Regulatory	343, 382	Regulatory	*PPP6R1* *PPP6R2*		
PP7	653		*PPM1G*		Retinitis Pigmentosa Retinoblastoma	[38]

## 2. Protein Phosphatases and Their Involvement in AD

### 2.1. Protein Phosphatase 1 (PP1)

PP1 is a crucial enzyme involved in the regulation of various cellular processes, including cell division, glycogen metabolism, muscle contraction, and the neuronal signaling pathways [39]. PP1 has been implicated with AD in several aspects, including tau protein phosphorylation, amyloid precursor protein (APP) processing, synaptic dysfunction, neuronal survival, and therapeutic targeting, respectively [40]. The dysregulation of PP1 activity or localization may lead to the hyperphosphorylation of tau, disrupting microtubule stability and contributing to the formation of NFTs, a pathological hallmark of AD [41]. Furthermore, it has also been observed that the dysregulation of PP1 activity may influence the cleavage of APP by secretases, leading to the altered production of amyloid-beta (Aβ) peptides, which results in the formation of amyloid plaques and leads to AD [20,42]. PP1 is also linked to the regulation of cell survival pathways, and altered PP1 activity may disrupt the signaling pathways implicated in neuronal survival and apoptosis, which may contribute to the neuronal loss observed in AD [43]. 

Moreover, PP1 is also linked to ionic conductance, long-term synaptic plasticity, [44] and dephosphorylating substrates in postsynaptic densities (PSDs) associated with Ca^2+^/calmodulin-dependent protein kinase-II (CaMKII) [45]. Mammalian PP1s are particularly inhibited by the heat-stable inhibitors I-1 and I-2 and preferentially dephosphorylate the subunit of phosphorylase kinase [46]. Long-term potentiation (LTP) and long-term depression (LTD) are two neuronal processes that underpin learning and memory, and they are regulated by PP1 through synaptic plasticity [47]. Therefore, modified PP1 activity results in changes in the phosphorylation status of synaptic proteins that is essential for the induction, maintenance, and reversal of synaptic plasticity [48].

**Figure 1:**
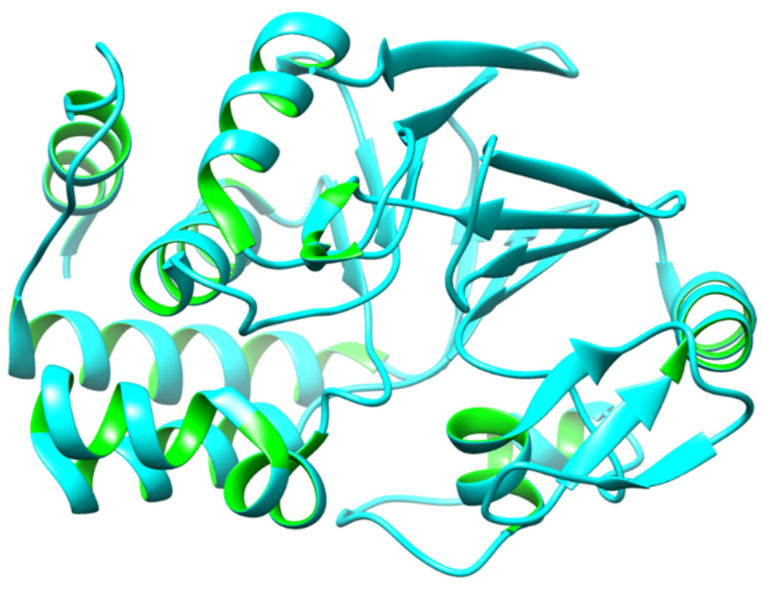
The 3D protein structure of PP1.

Additionally, PP1 is also connected to AD through the hyperphosphorylation of microtubule-associated proteins (MAPs) in the brain. Moreover, it has been found that there is significantly lower PP1 activity in both gray and white matters in AD brains, which clearly demonstrates that dysfunctional phosphatases play a significant role in AD [49]. In contrast to PP1, PP2As are insensitive to I-1 and I-2 and dephosphorylate the phosphorylase kinase subunit [50]. By examining PP1 as a single entity, we can see that the PP1 holoenzyme is composed of a catalytic subunit (PP1c) and one (or occasionally two) regulatory (R) subunit. These subunits comprise a huge collection of over 200 potential PP1c interactors [51]. However, to put it another way, PP1 should be investigated as a crucial component of a vast and changeable interactome (a complex representation of the functional interactions between molecules within a cell or within an organism). Through the comprehension of the PP1 interactome, its suitable role in the etiology of heart failure becomes visible [52]. The 3D protein model is depicted in Figure 1. 

### 2.2. Protein Phosphatase 2A (PP2A)

**Figure 2:**
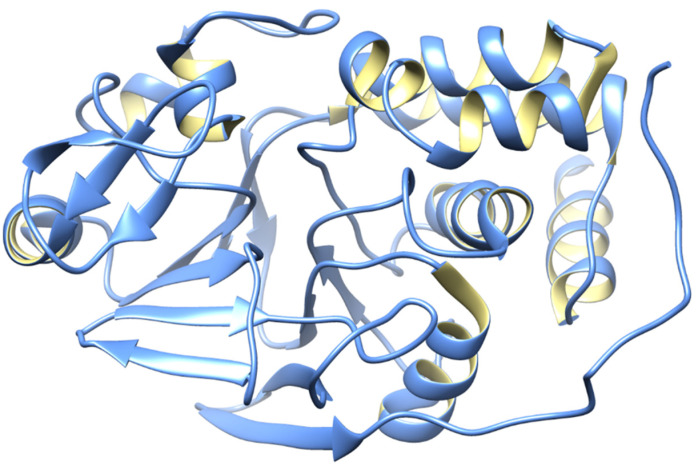
3D structure of PP2A.

The *PPP2CA* in humans encodes the enzyme protein phosphatase 2 (PP2), commonly known as PP2A [53]. The heterotrimeric PP2A is widely expressed and contributes significantly to phosphatase activity in eukaryotic cells [54]. Many cellular processes are carried out by its serine/threonine phosphatase activity with substrate specificity. The published data show that the targets of PP2A are proteins of oncogenic signaling cascades, such as Raf, Mitogen-activated protein kinase (MEK), and AKT, with PP2A acting as a tumor suppressor [55]. The structure of PP2A is shown in Figure 2. 

PP2A is an evolutionarily conserved enzyme that regulates the majority of signal transduction pathways and physiological functions [56,57]. The mammalian PP2A holoenzyme is a heterotrimer made up of catalytic (C or PPP2C), structural (A or PPP2R1), and regulatory (B-type) subunits [57]. There are four known families of regulatory “B-type” subunits, including PPP2R2, PPP2R5, PPP2R3, and PPP2R6, and two isoforms of the A and C subunits (α and β). A highly complicated and currently little understood mechanism that ensures PP2A substrate specificity is the control of PP2A biogenesis, activity, and targeting [58]. They are partially regulated by the binding of a certain B subunit and other regulators to the PP2A (AC) core protein, in addition to post-translational alterations (methylation, phosphorylation, and ubiquitination) of the catalytic domain [59]. The particular targeting of PP2A by several pathogenic viruses and parasites serves as an illustration of the crucial role that PP2A plays in cell signaling and homeostasis [60]. Based on the critical role which the PP2A family plays in the control of key cellular processes, it is not surprising that PP2A malfunction is linked to human disorders, such as neurodegenerative diseases [61], heart disease, diabetes [62], asthma [63], and cancer [57]. Numerous human tumorigeneses have altered PP2A subunit expression. For instance, the PP2A Cα [64] and Bα [65] subunits are downregulated in prostate cancer, whereas breast and lung carcinomas have a downregulated A subunit [66]. Furthermore, CIP2A and SET, the endogenous PP2A inhibitors, are elevated in several malignancies [67]. As a result, PP2A has been believed to be a tumor suppressor [68]. However, in certain malignancies, the expression of PP2A subunits is also increased. For instance, pancreatic cancer has increased Bα levels that support oncogenic activation and encourage metastasis [69]. 

**Figure 3:**
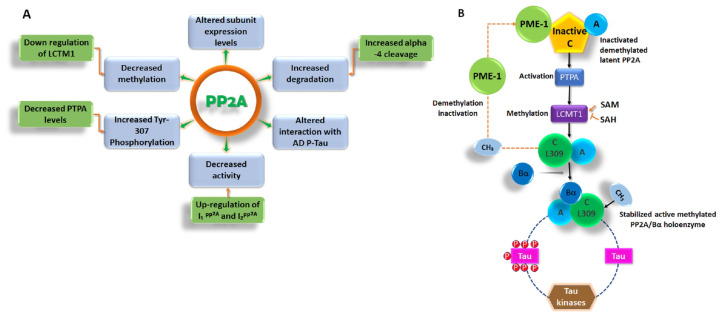
Overview of PP2A dysfunction in AD and its link to the deregulation of tau. (**A**) Altered PP2A subunit expression, activity, and post-translational modifications have been described in AD autopsy brain tissues. Some of these changes may be mediated by alterations in specific PP2A modulatory proteins (LCMT1, PTPA, alpha4) and endogenous PP2A inhibitors (I1PP2A and I2PP2A) that have also been reported in AD autopsy brain tissues. They also decrease the interaction of PP2A with tau. (**B**) The biogenesis of the PP2A/Bα holoenzyme, the primary Ser/Thr tau phosphatase in vivo, is believed to be controlled by the Leu-309 methylation of the PP2A catalytic subunit by LCMT1. This reaction requires a supply of SAM, the universal methyl donor, and is inhibited by SAH. The PP2A methylesterase, PME-1, can demethylate and inactivate PP2A through distinct mechanisms and form a complex with inactive PP2A enzymes. These inactive complexes could be-reactivated via the action of the PP2A activator PTPA, allowing for the subsequent methylation of the PP2A C subunit. Many brain Ser/Thr protein kinases, including GSK3β, oppose the action of PP2A/Bα and promote tau phosphorylation. The inhibition and/or downregulation of PP2A can enhance tau phosphorylation directly, by preventing its dephosphorylation, or indirectly, by upregulating tau kinases.

Moreover, PP2 is considered a good therapeutic and biological target to discover new chemical scaffolds and inhibitors against Parkinson’s disease (PD) and AD, respectively [61,70]. Experimental data have clearly depicted PP2A disfunction as a main pillar in the progress of tau pathology in AD [61]. Furthermore, PP2A also opposes the activity of many brain protein kinases upregulated in AD. Therefore, developing PP2A-targeted therapies for AD particularly against the P-tau pathology could be highly significant in treating AD. In this regard, multiple chemical compounds have been tested to regulate/alter PP2A functionality by different mechanisms through direct or indirect ways [71]. Figure 3 provides an overview of PP2A dysfunction in AD and shows its linking to the deregulation of tau.

### 2.3. Protein Phosphatase 2B (PP2B)

**Figure 4:**
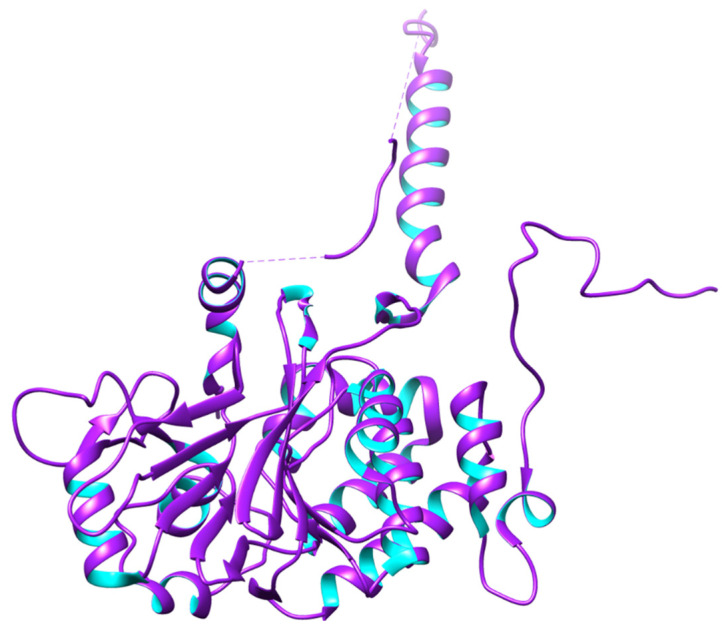
3D structure of PP2B. PP2B is associated with AD through the dephosphorylation of the tau protein at different phosphorylation sites, and it has also been observed that PP2B activity is either unchanged or decreased in the AD brain. Based on these speculations, PP2B might regulate tau phosphorylation, and a downregulation of PP2B might contribute to the abnormal hyperphosphorylation of tau [80,81].

In the brain, PP2B is known as calcineurin and is a Ca^2+^/calmodulin-dependent protein phosphatase. The holoenzyme is a heterodimer made up of an 18-kDa regulatory subunit (B-subunit) and a 60-kDa catalytic subunit (A-subunit) [72]. The calmodulin-binding domain and the autoinhibitory domain in the catalytic subunit’s C-terminal region often hide the catalytic domain and maintain the enzyme’s inactive state [73]. Ca^2+^/calmodulin binding to the calmodulin-binding domain causes the autoinhibitory domain to be released from the catalytic site, activating PP2B [74]. The autoinhibitory domains of PP2B can also be activated by proteolytic cleavage, leading to a Ca^2+^/calmodulin-independent, activated phosphatase [75]. It has been reported that calpain I, a major Ca^2+^-activated protease in the brain, cleaves and activates PP2B [75,76]. PP2B is one of the most abundant phosphatases in the brain [77] and may be generated by combining two regulatory B-subunit isoforms (type-1/2) with any of the three catalytic A-subunit isoforms (α, β, and γ) [78]. These isoforms lack the targeted PP1 holoenzymes’ structural and functional variety; therefore, the many functions of PP2B in biological processes are frequently mediated by a similarly broad spectrum of interacting proteins. However, most of these interactions happen between two different PP2B surfaces. A β-sheet on the catalytic subunit makes up the surface that has been better defined. The PxIxIT motif, a conserved motif, interacts with this β-sheet. The PxIxIT motif creates a brief β-strand that interacts with the catalytic subunit’s β-strand 14 and lengthens the β-sheet [79]. The predicted structure of PP2B is shown in Figure 4.

### 2.4. Protein Phosphatase 3 (PP3)

**Figure 5:**
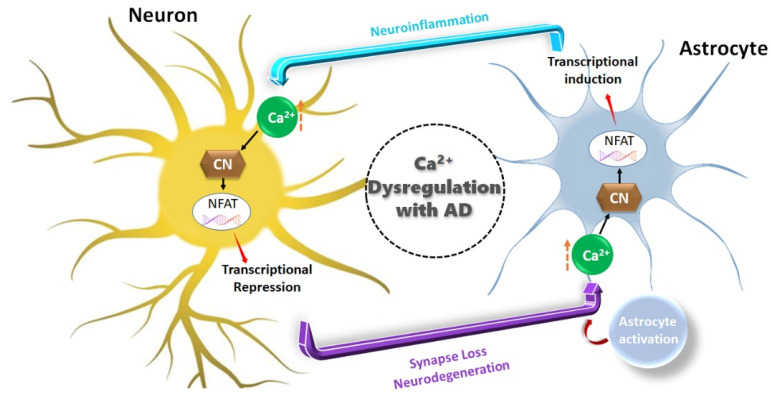
Ca^2+^ dysregulation is widespread in neurons and astrocytes in animal models of AD because of amyloid pathology and cellular damage, among many other possible causes. CN is very susceptible to Ca^2+^ dysregulation and demonstrates increased activity in both neurons and astrocytes with AD. The downregulation of neuronal genes by CN results in synaptic loss, neurite degeneration, and decreased neuronal survival, which activates adjacent astrocytes, resulting in astrocytic Ca^2+^ dysregulation and astrocytic hyperactivity, which lead to cognitive decline and dementia.

PP3 is also known as calcineurin (CaN), the only serine/threonine protein phosphatase under the control of Ca^2+^/calmodulin, and performs a crucial part in the connection between Ca^2+^ signals and cellular reactions in the body [54]. Several tissues, including the brain, heart, kidney, liver, muscle, eye, and T-lymphocytes, have been shown to possess CaN [54,82]. The hypertrophic growth of cardiac and skeletal muscle in response to mechanical stress is also dependent on Ca^2+^ signaling [83]. Another study has showed that immunosuppressive medications such as cyclosporin A (lipophilic cyclic polypeptide) and tacrolimus (FK506) control the activity of calmodulin-dependent phosphatase calcineurin through their interaction with cyclophilin and FKBP12, respectively [84]. Therefore, it has also been observed that CaN mediates or exacerbates AD pathophysiology through the activation of the nuclear factor of the activated T cell (NFAT) family of transcription factors [85]. Mouse studies have explored the signaling cascade of CaN/NFAT in hippocampal neurons with increased CaN expression/activity, which plays a significant role in transcriptional suppression. Moreover, CaN/NFAT in astrocyte signaling is associated with neurodegeneration and AD through Ca^2+^ dysregulation [86] (see Figure 5). 

### 2.5. Protein Phosphatase 4 (PP4)

**Figure 6:**
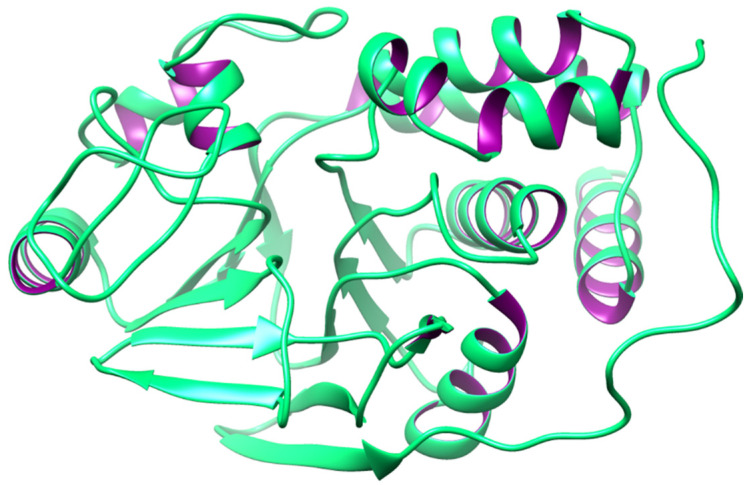
The overall structure of PP4.

PP4, also known as PPX, is allegedly involved in mammalian microtubule centrosome interaction control [87]. PP4 is a complex protein composed of one catalytic subunit (PP4C) and five regulatory subunits, such as PP4R1, PP4R2, PP4R3α, PP4R3β, and PP4R4, respectively [88] (Figure 6). The phosphorylation status of tau and APP proteins is regulated by PP4, either directly or indirectly. This affects both proteins’ processing and aggregation, which are crucial to the pathophysiology of AD. PP4 is also linked to different signaling pathways such as PI3K/Akt, MAPK/ERK, and the Wnt/β-catenin pathway, which have been implicated in AD [89]. Therefore, the dysregulation of these pathways has been associated with synaptic dysfunction, neuroinflammation, and neuronal cell death and causes AD. These facts show the important role of PP4 in modulating AD-related signaling cascades. 

There are multiple reports that show the functional role of PP4 in the survival of the motor neuron complex, spliceosome assembly, and neuron disorders [90]. The survival motor neuron (SMN) protein is typically altered in the neurodegenerative disorder called spinal muscular atrophy (SMA) [91]. Small nuclear ribonucleoproteins (snRNPs) and small nucleolar ribonucleoproteins (snoRNPs) are assembled in the cytoplasm, transported to the nucleus, and matured in nuclear bodies known as cajal or coiled bodies by these SMN multi-protein complexes [91]. PP4 is structurally and functionally linked to PP2A and adheres to the same general assembly and regulatory principles as PP2A [92]. PP4 can form both heterodimers and heterotrimers [93]. PP4 is composed of six regulatory subunits [94]. This phosphatase has established the idea of substrate selectivity imparted by PP4 regulatory subunits. The phosphorylated histone 2A variant H2AX, which is a marker of DNA damage and cell cycle arrest, is dephosphorylated by PP4, and, during the S phase, PP4 functionally regulates H2AX phosphatase activity [95]. During DNA replication, a particular PP4 heterotrimeric complex, including the catalytic subunit (PP4C), the scaffolding component PP4R2, and the targeting subunit PP4R3, restores H2AX to its dephosphorylated form [96]. Other PP4 regulatory genes, such as PP4R1 or PP4R3, however, have shown no influence on -H2AX dephosphorylation. More importantly, PP4 has a direct association with the Toll-like receptor (TLR) signaling pathways [97]. Therefore, it is also well known that TLR4-mediated signaling is correlated with the pathogenesis of age-related neurodegenerative diseases, particularly AD [98]. 

### 2.6. Protein Phosphatase 5 (PP5)

**Figure 7:**
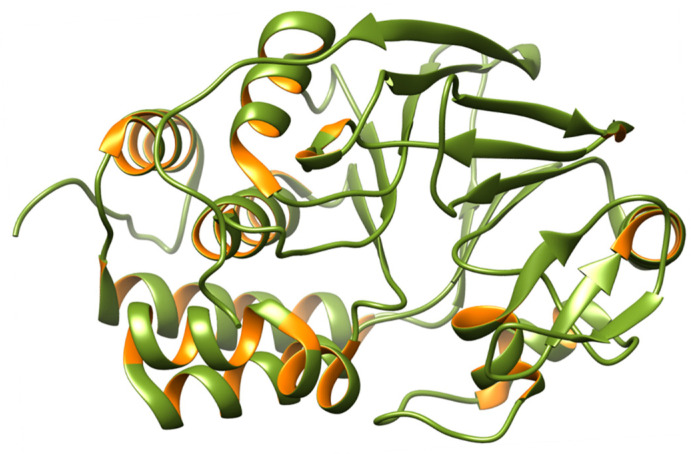
The overall structure of PP5.

PP5 is a 58-kDa phosphoseryl/phosphothreonyl protein that is distinct from other serine/threonine phosphatases because it contains tetratrico-peptide repeat (TPR) domains. PP5 is highly expressed in the mammalian brain and has been involved in multiple cellular processes, such as MAPK-mediated growth and differentiation, cell cycle arrest, and ATM/ATR pathways [99]. It has been observed that PP5 dephosphorylates the tau protein in AD. Cell line studies show that the overexpression of PP5 in PC12 cells results in the dephosphorylation of tau at multiple phosphorylation sites [100]. In the AD neocortex, PP5 activity has been shown to be reduced by about 20%. These results have shown that tau is most likely a physiological substrate of PP5, and the aberrant hyperphosphorylation of tau in AD is due to diminished PP5 activity in the diseased brains. The in vitro results show that PP5 dephosphorylates tau and that it may connect with microtubules, suggesting that it may possibly be involved in modulating tau phosphorylation in the brain [101]. Furthermore, this study also suggested that tau phosphorylation may be regulated by PP5, and tau hyperphosphorylation in AD may be caused by decreasing PP5 activity [100,101]. Moreover, PP5 can act as a neuroprotectant to lessen the negative consequences of Aβ toxicity [36]. Past studies have indicated that Aβ damages mitochondrial activity and raises the levels of ROS, which may be directly responsible for neuronal toxicity [102]. In cortical neurons in culture, increased Aβ-induced cell death has been linked to PP5 downregulation, whereas PP5 overexpression has the opposite impact. PP5 may be able to counteract the effect of Aβ through its capability to inhibit the MAP kinase pathways implicated in apoptosis [103]. The 3D protein structure of PP5 is shown in Figure 7.

### 2.7. Protein Phosphatase 6 (PP6)

**Figure 8:**
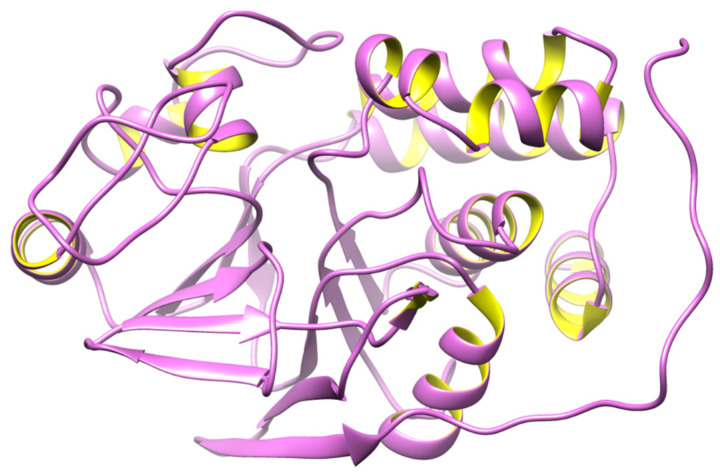
3D structure of PP6.

The PP6 holoenzyme is a heterotrimeric complex formed by the catalytic subunit and an ankyrin repeat domain-containing regulatory subunit (ARS) [104]. It has also been observed that C-terminal methylation influences PP6 holoenzyme composition and that variations in holoenzyme assembly are associated with AD [103]. The catalytic subunit of PP6 is involved in the signaling pathway and cell cycle progression in response to IL2 receptor stimulation [105,106,107]. The N-terminus domain inhibits G1-to-S phase progression in cancer cells, in part through the control of cyclin D1 [108]. Moreover, in the mitosis process, it regulates spindle positioning [109]. Through dephosphorylating MAP3K7, PP6 inhibits the MAP3K7 kinase activation of the IL1 signaling pathway [105]. Prior data have also shown that specified polymorphisms in the genes encoding the α and β isoforms of interleukin-l (IL-1) are linked to an increased risk of AD. The overexpression of IL-1 is linked to the development of β-amyloid plaque, and higher IL-1 levels have been seen in Alzheimer’s brains. Furthermore, βAPP, apolipoprotein E, α1-antichymotrypsin, and α2-macroglobulin are a few of the more recognized or speculated genetic risk factors for AD that interact with IL-1. Moreover, IL-1 overexpression is linked to environmental risk variables for AD, such as aging naturally and head trauma. These findings imply that IL-1 and IL-1-driven networks play a significant pathogenic role in the development of AD [110]. The structure of PP6 is shown in Figure 8.

### 2.8. Protein Phosphatases 7 (PP7)

**Figure 9:**
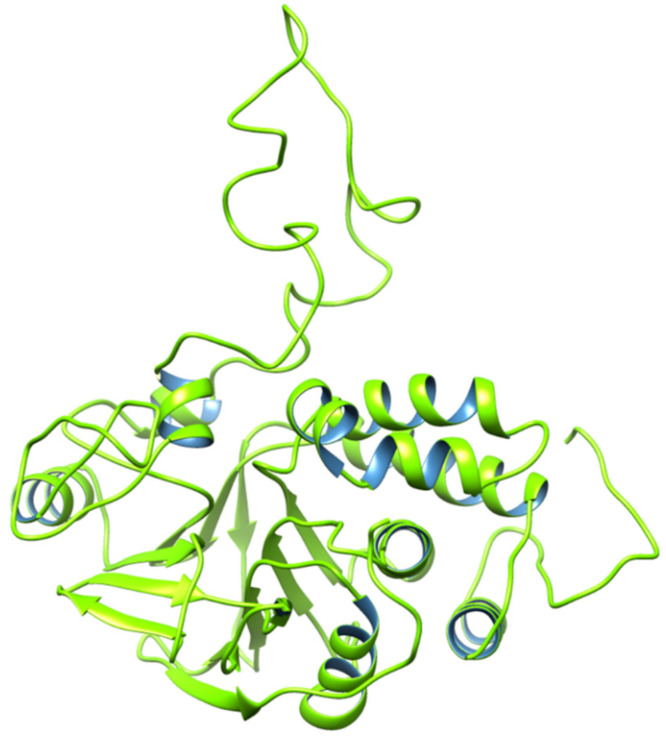
3D structure of PP7.

PP7 is another protein consisting of 653 amino acids, with a predicted molecular mass of ~75 kDa, which belongs to the superfamily of protein phosphatases (PPs). Structural data have shown that EF-hand and EF-hand-like motifs are five potential high-affinity calcium-binding domains in an extensive C-terminal region of protein PP7. PP7 is involved in various cellular processes, including cell cycle regulation, transcription, DNA repair, and stress response, respectively [111]. Moreover, disruption in these PP7-regulated cellular activities may play a significant role in the pathophysiology of AD, since they are essential for preserving neuronal health. Additionally, PP7 is linked to AD through tau phosphorylation, Aβ toxicity, and neuroinflammation, respectively, but more studies are needed to completely understand PP7’s role in AD. The overall protein structure of PP7 is shown in Figure 9.

## 3. Pharmacological Agents Directly Targeting PP Receptors

There is ongoing research on the potential use of protein phosphatase inhibitors as a treatment for AD, but, currently, there are no FDA-approved AD drugs that target protein phosphatases. However, below is a list of some of the commercially available drugs that have been studied for their effects on protein phosphatases and their potential use in treating AD.

### 3.1. Lithium 

The activity of PP2A, which has been linked to the pathophysiology of AD, has been demonstrated to be inhibited by this drug [112,113]. Lithium is studied as a potential treatment for the cognitive symptoms of AD [112,114].

### 3.2. Okadaic Acid (OKA)

OKA is not a drug, but it is one of the most common polyether toxins generated by marine microalgae, responsible for diarrhetic shellfish poisoning. It induces tau hyperphosphorylation both in vitro and in vivo and is a selective and powerful inhibitor of serine/threonine phosphatases 1 and 2A. [115]. OKA is a potent inhibitor against PPs and has been employed in research studies to investigate the role of PP2A in AD [115,116].

### 3.3. Cantharidin, Cyclosporine A, and Tideglusib 

Cantharidin is a natural compound and a potent inhibitor of PP2A and has been studied for its potential therapeutic effects in AD [117]. Cyclosporine A, which is used primarily as an immunosuppressant, has been shown to inhibit the activity of calcineurin, a protein phosphatase which is involved in AD [118]. Furthermore, there are some experimental drugs that are being studied for their effects on protein phosphatases in AD. Tideglusib is a glycogen synthase kinase 3 (GSK-3) inhibitor that increases the activity of PP2A, which is reduced in the AD brain. It is currently in phase II clinical trials for AD [119]. 

### 3.4. CIGB-300, Anle138b, LB-100, and Salubrinal

CIGB-300 is a peptide that targets the interaction between PP2A and tau, a protein which forms tangles in the brains of people with AD. It has shown promising results in preclinical studies and is currently in phase I clinical trials [120,121]. Anle138b, a drug which targets the aggregation of tau, has been shown to increase the activity of PP2A in preclinical studies [122]. LB-100 is another drug that is effective as a PP2A inhibitor and has been demonstrated to be promising in preclinical research for the treatment of AD [123,124]. Similarly, Salubrinal is a drug that inhibits a specific protein phosphatase, eIF2α phosphatase, and has been shown to improve cognitive function in preclinical studies of AD [125].

### 3.5. AV-1451 and Fingolimod 

AV-1451, also known as flortaucipir, is a positron emission tomography (PET) imaging agent that can detect tau protein deposits in the brain [126]. While not a drug in itself, AV-1451 is being used in clinical trials to assess the effects of potential tau-targeting drugs on protein phosphatases in the brains of people with AD [127]. Fingolimod, a drug which is currently used to treat multiple sclerosis, has been shown to increase the activity of PP2A and reduce the accumulation of amyloid beta, a protein which forms plaques in the brains of people with AD [128]. It is currently in the early stage of clinical trials for AD [129]. Again, it is important to note that these drugs are still in the experimental stages, and more research is needed to determine their safety and efficacy for the treatment of AD. Clinical trials are ongoing to further evaluate these drugs, and it may take several years before any of them are approved for use in treating AD.

## 4. Conclusions and Future Prospects

In this review, we discussed various PPs and highlighted their significant role in brain-related diseases such as AD. Collectively, PP1-PP7 have direct and indirect influences on AD through the activation and deactivation of downstream signaling pathways. Overall, our knowledge about the critical role of phosphatases in neurodegeneration is constantly growing, and, additionally, there is urgent need for research identifying changes in PP expression, activity, or mutation that affect AD onset and progression. Therefore, new connections to human illness are being revealed, and advancements in understanding PP function are being made. It is feasible that significant information about treatment development may emerge either from targets inside the pathways they modulate or from protein phosphatases directly. In the future, attention should be given to phosphatase-targeted therapies for neurodegenerative disorders, and we hope that this will open promising new avenues for developing effective drugs. 

## References

[1] Seok S.-H. (2021). Structural insights into protein regulation by phosphorylation and substrate recognition of protein kinases/phosphatases. Life.

[2] Choy M.S., Hieke M., Kumar G.S., Lewis G.R., Gonzalez-DeWhitt K.R., Kessler R.P., Stein B.J., Hessenberger M., Nairn A.C., Peti W. (2014). Understanding the antagonism of retinoblastoma protein dephosphorylation by PNUTS provides insights into the PP1 regulatory code. Proc. Natl. Acad. Sci. USA.

[3] Lee M.J., Yaffe M.B. (2016). Protein Regulation in Signal Transduction. Cold Spring Harb. Perspect. Biol..

[4] Manning G., Plowman G.D., Hunter T., Sudarsanam S. (2002). Evolution of protein kinase signaling from yeast to man. Trends Biochem. Sci..

[5] Cohen P.T. (1997). Novel protein serine/threonine phosphatases: Variety is the spice of life. Trends Biochem. Sci..

[6] Denu J.M., Dixon J.E. (1998). Protein tyrosine phosphatases: Mechanisms of catalysis and regulation. Curr. Opin. Chem. Biol..

[7] Janssens V., Goris J. (2001). Protein phosphatase 2A: A highly regulated family of serine/threonine phosphatases implicated in cell growth and signalling. Biochem. J..

[8] Ceulemans H., Bollen M. (2004). Functional diversity of protein phosphatase-1, a cellular economizer and reset button. Physiol. Rev..

[9] Honkanen R., Golden T. (2002). Regulators of serine/threonine protein phosphatases at the dawn of a clinical era?. Curr. Med. Chem..

[10] Li R., Liu Y. (2016). Physical activity and prevention of Alzheimer’s disease. J. Sport Health Sci..

[11] Zissimopoulos J., Crimmins E., St. Clair P. (2015). The value of delaying Alzheimer’s disease onset. Forum Health Econ. Policy.

[12] Werner P. (2023). Like beauty and contact lenses, the meaning of dementia behavioral changes is in the eyes of the beholder. Int. Psychogeriatr..

[13] Insel K.C., Badger T.A. (2002). Deciphering the 4 D’s: Cognitive decline, delirium, depression and dementia–a review. J. Adv. Nurs..

[14] Moustafa A.A., Hassan M., Hewedi D.H., Hewedi I., Garami J.K., Al Ashwal H., Zaki N., Seo S.-Y., Cutsuridis V., Angulo S.L. (2018). Genetic underpinnings in Alzheimer’s disease–a review. Rev. Neurosci..

[15] Isik A.T. (2010). Late onset Alzheimer’s disease in older people. Clin. Interv. Aging.

[16] Wang J.-Z., Wang Z.-H., Tian Q. (2014). Tau hyperphosphorylation induces apoptotic escape and triggers neurodegeneration in Alzheimer’s disease. Neurosci. Bull..

[17] Zhang S., Wang Z., Cai F., Zhang M., Wu Y., Zhang J., Song W. (2017). BACE1 cleavage site selection critical for amyloidogenesis and Alzheimer’s pathogenesis. J. Neurosci..

[18] Gong C.X., Shaikh S., Wang J.Z., Zaidi T., Grundke-Iqbal I., Iqbal K. (1995). Phosphatase activity toward abnormally phosphorylated τ: Decrease in Alzheimer disease brain. J. Neurochem..

[19] Wang J.-z., Wu Q., Smith A., Grundke-Iqbal I., Iqbal K.J.F.l. (1998). τ is phosphorylated by GSK-3 at several sites found in Alzheimer disease and its biological activity markedly inhibited only after it is prephosphorylated by A-kinase. FEBS Lett..

[20] Vintém A.P.B., Henriques A.G., e Silva O.A.d.C., e Silva E.F.d.C. (2009). PP1 inhibition by Aβ peptide as a potential pathological mechanism in Alzheimer’s disease. Neurotoxicol. Teratol..

[21] Cankara F.N., Kuş M.S., Günaydın C., Şafak S., Bilge S.S., Ozmen O., Tural E., Kortholt A. (2022). The beneficial effect of salubrinal on neuroinflammation and neuronal loss in intranigral LPS-induced hemi-Parkinson disease model in rats. Immunopharmacol. Immunotoxicol..

[22] Lontay B., Kiss A., Virág L., Tar K. (2020). How do post-translational modifications influence the pathomechanistic landscape of Huntington’s disease? A comprehensive review. Int. J. Mol. Sci..

[23] Li M.-L., Peng Y., An Y., Li G.-Y., Lan Y. (2022). LY395756 promotes NR2B expression via activation of AKT/CREB signaling in the juvenile methylazoxymethanol mice model of schizophrenia. Brain Behav..

[24] Leslie S.N., Nairn A.C. (2019). cAMP regulation of protein phosphatases PP1 and PP2A in brain. Biochim. Biophys. Acta (BBA)-Mol. Cell Res..

[25] Wei H., Zhang H.-l., Wang X.-c., Xie J.-z., An D.-d., Wan L., Wang J.-z., Zeng Y., Shu X.-j., Westermarck J. (2020). Direct activation of protein phosphatase 2A (PP2A) by tricyclic sulfonamides ameliorates Alzheimer’s disease pathogenesis in cell and animal models. Neurotherapeutics.

[26] Su J., Zhang J., Bao R., Xia C., Zhang Y., Zhu Z., Lv Q., Qi Y., Xue J. (2021). Mitochondrial dysfunction and apoptosis are attenuated through activation of AMPK/GSK-3β/PP2A pathway in Parkinson’s disease. Eur. J. Pharmacol..

[27] Javadpour P., Dargahi L., Ahmadiani A., Ghasemi R. (2019). To be or not to be: PP2A as a dual player in CNS functions, its role in neurodegeneration, and its interaction with brain insulin signaling. Cell. Mol. Life Sci..

[28] Di Domenico F., Tramutola A., Barone E., Lanzillotta C., Defever O., Arena A., Zuliani I., Foppoli C., Iavarone F., Vincenzoni F. (2019). Restoration of aberrant mTOR signaling by intranasal rapamycin reduces oxidative damage: Focus on HNE-modified proteins in a mouse model of down syndrome. Redox Biol..

[29] Caberlotto L., Nguyen T.-P. (2014). A systems biology investigation of neurodegenerative dementia reveals a pivotal role of autophagy. BMC Syst. Biol..

[30] Hofstetter C.P., Burkhardt J.-K., Shin B.J., Gürsel D.B., Mubita L., Gorrepati R., Brennan C., Holland E.C., Boockvar J.A. (2012). Protein phosphatase 2A mediates dormancy of glioblastoma multiforme-derived tumor stem-like cells during hypoxia. PLoS ONE.

[31] Martin L., Latypova X., Wilson C.M., Magnaudeix A., Perrin M.-L., Terro F. (2013). Tau protein phosphatases in Alzheimer’s disease: The leading role of PP2A. Ageing Res. Rev..

[32] Anantharam V., Lehrmann E., Kanthasamy A., Yang Y., Banerjee P., Becker K.G., Freed W.J., Kanthasamy A.G. (2007). Microarray analysis of oxidative stress regulated genes in mesencephalic dopaminergic neuronal cells: Relevance to oxidative damage in Parkinson’s disease. Neurochem. Int..

[33] Sawant N., Reddy P.H. (2019). Role of phosphorylated Tau and glucose synthase kinase 3 beta in Huntington’s disease progression. J. Alzheimer’s Dis..

[34] Ohama T. (2019). The multiple functions of protein phosphatase 6. Biochim. Biophys. Acta (BBA)-Mol. Cell Res..

[35] Zhang H.-L., Wang X.-C., Liu R. (2022). Zinc in Regulating Protein Kinases and Phosphatases in Neurodegenerative Diseases. Biomolecules.

[36] Sanchez-Ortiz E., Hahm B.K., Armstrong D.L., Rossie S. (2009). Protein phosphatase 5 protects neurons against amyloid-beta toxicity. J. Neurochem..

[37] Kitamura N., Fujiwara N., Hayakawa K., Ohama T., Sato K. (2021). Protein phosphatase 6 promotes neurite outgrowth by promoting mTORC2 activity in N2a cells. J. Biochem..

[38] Schmidt K. (2004). Analysis of the Structure and Function of Protein Phosphatase 2A. Ph.D. Thesis.

[39] Wang B., Zhang P., Wei Q. (2008). Recent progress on the structure of Ser/Thr protein phosphatases. Sci. China Ser. C Life Sci..

[40] Govindarajulu M., Pinky P.D., Bloemer J., Ghanei N., Suppiramaniam V., Amin R. (2018). Signaling mechanisms of selective PPARγ modulators in Alzheimer’s disease. PPAR Res..

[41] Liu F., Grundke-Iqbal I., Iqbal K., Gong C.X. (2005). Contributions of protein phosphatases PP1, PP2A, PP2B and PP5 to the regulation of tau phosphorylation. Eur. J. Neurosci..

[42] da Cruz e Silva E.F., da Cruz e Silva O.A., Zaia C.T.B., Greengard P. (1995). Inhibition of protein phosphatase 1 stimulates secretion of Alzheimer amyloid precursor protein. Mol. Med..

[43] Perluigi M., Barone E., Di Domenico F., Butterfield D. (2016). Aberrant protein phosphorylation in Alzheimer disease brain disturbs pro-survival and cell death pathways. Biochim. Biophys. Acta (BBA)-Mol. Basis Dis..

[44] Belmeguenai A., Hansel C. (2005). A role for protein phosphatases 1, 2A, and 2B in cerebellar long-term potentiation. J. Neurosci..

[45] Ishida A., Shigeri Y., Taniguchi T., Kameshita I. (2003). Protein phosphatases that regulate multifunctional Ca^2+^/calmodulin-dependent protein kinases: From biochemistry to pharmacology. Pharmacol. Ther..

[46] Huang K.X., Paudel H.K. (2000). Ser67-phosphorylated inhibitor 1 is a potent protein phosphatase 1 inhibitor. Proc. Natl. Acad. Sci. USA.

[47] Antunes G., Roque A.C., Simoes-de-Souza F. (2016). Stochastic induction of long-term potentiation and long-term depression. Sci. Rep..

[48] Foley K., McKee C., Nairn A.C., Xia H. (2021). Regulation of synaptic transmission and plasticity by protein phosphatase 1. J. Neurosci..

[49] Gong C.X., Singh T.J., Grundke-Iqbal I., Iqbal K. (1993). Phosphoprotein phosphatase activities in Alzheimer disease brain. J. Neurochem..

[50] Farkas I., Dombradi V., Miskei M., Szabados L., Koncz C. (2007). Arabidopsis PPP family of serine/threonine phosphatases. Trends Plant Sci..

[51] Chiang D.Y., Heck A.J., Dobrev D., Wehrens X.H. (2016). Regulating the regulator: Insights into the cardiac protein phosphatase 1 interactome. J. Mol. Cell. Cardiol..

[52] Chiang D.Y., Alsina K.M., Corradini E., Fitzpatrick M., Ni L., Lahiri S.K., Reynolds J.O., Pan X., Scott Jr L., Heck A.J. (2018). Rearrangement of the protein phosphatase 1 interactome during heart failure progression. Circulation.

[53] Jones T., Barker H., e Silva E.d.C., Mayer-Jaekel R., Hemmings B., Spurr N., Sheer D., Cohen P. (1993). Localization of the genes encoding the catalytic subunits of protein phosphatase 2A to human chromosome bands 5q23→ q31 and 8p12→ p11. 2, respectively. Cytogenet. Genome Res..

[54] O’Donoghue J. (2006). Hydroquinone and its analogues in dermatology—A risk-benefit viewpoint. J. Cosmet. Dermatol..

[55] Sahab Z.J., Hall M.D., Zhang L., Cheema A.K., Byers S.W. (2010). Tumor suppressor rarres1 regulates dlg2, pp2a, vcp, eb1, and ankrd26. J. Cancer.

[56] Sontag E. (2001). Protein phosphatase 2A: The Trojan Horse of cellular signaling. Cell. Signal..

[57] Seshacharyulu P., Pandey P., Datta K., Batra S.K. (2013). Phosphatase: PP2A structural importance, regulation and its aberrant expression in cancer. Cancer Lett..

[58] Schuhmacher D., Sontag J.M., Sontag E. (2019). Protein Phosphatase 2A: More Than a Passenger in the Regulation of Epithelial Cell-Cell Junctions. Front. Cell Dev. Biol..

[59] Sents W., Ivanova E., Lambrecht C., Haesen D., Janssens V. (2013). The biogenesis of active protein phosphatase 2A holoenzymes: A tightly regulated process creating phosphatase specificity. FEBS J..

[60] Garcia A., Cayla X., Sontag E. (2000). Protein phosphatase 2A: A definite player in viral and parasitic regulation. Microbes Infect..

[61] Sontag J.-M., Sontag E. (2014). Protein phosphatase 2A dysfunction in Alzheimer’s disease. Front. Mol. Neurosci..

[62] Baskaran R., Velmurugan B.K. (2018). Protein phosphatase 2A as therapeutic targets in various disease models. Life Sci..

[63] Kobayashi Y., Mercado N., Barnes P.J., Ito K. (2011). Defects of protein phosphatase 2A causes corticosteroid insensitivity in severe asthma. PLoS ONE.

[64] Singh A.P., Bafna S., Chaudhary K., Venkatraman G., Smith L., Eudy J.D., Johansson S.L., Lin M.-F., Batra S.K. (2008). Genome-wide expression profiling reveals transcriptomic variation and perturbed gene networks in androgen-dependent and androgen-independent prostate cancer cells. Cancer Lett..

[65] Cheng Y., Liu W., Kim S.-T., Sun J., Lu L., Sun J., Zheng S.L., Isaacs W.B., Xu J. (2011). Evaluation of PPP2R2A as a prostate cancer susceptibility gene: A comprehensive germline and somatic study. Cancer Genet..

[66] Calin G.A., Di Iasio M.G., Caprini E., Vorechovsky I., Natali P.G., Sozzi G., Croce C.M., Barbanti-Brodano G., Russo G., Negrini M. (2000). Low frequency of alterations of the α (PPP2R1A) and β (PPP2R1B) isoforms of the subunit A of the serine-threonine phosphatase 2A in human neoplasms. Oncogene.

[67] Janghorban M., Farrell A.S., Allen-Petersen B.L., Pelz C., Daniel C.J., Oddo J., Langer E.M., Christensen D.J., Sears R.C. (2014). Targeting c-MYC by antagonizing PP2A inhibitors in breast cancer. Proc. Natl. Acad. Sci. USA.

[68] Mumby M. (2007). PP2A: Unveiling a reluctant tumor suppressor. Cell.

[69] Bryant J.-P., Levy A., Heiss J., Banasavadi-Siddegowda Y.K. (2021). Review of PP2A tumor biology and antitumor effects of PP2A inhibitor LB100 in the nervous system. Cancers.

[70] Braithwaite S.P., Voronkov M., Stock J.B., Mouradian M.M. (2012). Targeting phosphatases as the next generation of disease modifying therapeutics for Parkinson’s disease. Neurochem. Int..

[71] Voronkov M., Braithwaite S.P., Stock J.B. (2011). Phosphoprotein phosphatase 2A: A novel druggable target for Alzheimer’s disease. Future Med. Chem..

[72] Alothaid H., Aldughaim M.S.K., Alamri S.S., Alrahimi J.S.M., Al-Jadani S.H. (2021). Role of calcineurin biosignaling in cell secretion and the possible regulatory mechanisms. Saudi J. Biol. Sci..

[73] Swulius M.T., Waxham M. (2008). Ca^2+^/calmodulin-dependent protein kinases. Cell. Mol. Life Sci..

[74] Carruthers N.J., Stemmer P.M. (2008). Methionine oxidation in the calmodulin-binding domain of calcineurin disrupts calmodulin binding and calcineurin activation. Biochemistry.

[75] Liu F., Grundke-Iqbal I., Iqbal K., Oda Y., Tomizawa K., Gong C.-X. (2005). Truncation and activation of calcineurin A by calpain I in Alzheimer disease brain. J. Biol. Chem..

[76] Baudry M., Chou M.M., Bi X. (2013). Targeting calpain in synaptic plasticity. Expert Opin. Ther. Targets.

[77] Liu F., Liang Z., Gong C. (2006). Hyperphosphorylation of tau and protein phosphatases in Alzheimer disease. Panminerva Medica.

[78] Kashani E., Vassella E. (2022). Pleiotropy of PP2A Phosphatases in Cancer with a Focus on Glioblastoma IDH Wildtype. Cancers.

[79] Nygren P.J., Scott J.D. (2016). Regulation of the phosphatase PP2B by protein–protein interactions. Biochem. Soc. Trans..

[80] Qian W., Yin X., Hu W., Shi J., Gu J., Grundke-Iqbal I., Iqbal K., Gong C.-X., Liu F. (2011). Activation of protein phosphatase 2B and hyperphosphorylation of Tau in Alzheimer’s disease. J. Alzheimer’s Dis..

[81] Tian Q., Wang J. (2002). Role of serine/threonine protein phosphatase in Alzheimer’s disease. Neurosignals.

[82] Lakshmikuttyamma A., Selvakumar P., Kakkar R., Kanthan R., Wang R., Sharma R.K. (2003). Activation of calcineurin expression in ischemia-reperfused rat heart and in human ischemic myocardium. J. Cell. Biochem..

[83] Olson E.N., Williams R.S. (2000). Remodeling muscles with calcineurin. Bioessays.

[84] Liu J., Farmer Jr J.D., Lane W.S., Friedman J., Weissman I., Schreiber S.L. (1991). Calcineurin is a common target of cyclophilin-cyclosporin A and FKBP-FK506 complexes. Cell.

[85] Taglialatela G., Rastellini C., Cicalese L. (2015). Reduced incidence of dementia in solid organ transplant patients treated with calcineurin inhibitors. J. Alzheimer’s Dis..

[86] Norris C.M. (2018). Calcineurin: Directing the damage in Alzheimer disease: An Editorial for ‘Neuronal calcineurin transcriptional targets parallel changes observed in Alzheimer disease brain’on page 24. J. Neurochem..

[87] Sumiyoshi E., Sugimoto A., Yamamoto M. (2002). Protein phosphatase 4 is required for centrosome maturation in mitosis and sperm meiosis in *C. elegans*. J. Cell Sci..

[88] Lee D.-H., Chowdhury D. (2011). What goes on must come off: Phosphatases gate-crash the DNA damage response. Trends Biochem. Sci..

[89] Yeh P.Y., Yeh K.-H., Chuang S.-E., Song Y.C., Cheng A.-L. (2004). Suppression of MEK/ERK signaling pathway enhances cisplatin-induced NF-κB activation by protein phosphatase 4-mediated NF-κB p65 Thr dephosphorylation. J. Biol. Chem..

[90] Cohen P.T., Philp A., Vázquez-Martin C. (2005). Protein phosphatase 4–from obscurity to vital functions. FEBS Lett..

[91] Melki J. (1997). Spinal muscular atrophy. Curr. Opin. Neurol..

[92] Kloeker S., Wadzinski B.E. (1999). Purification and identification of a novel subunit of protein serine/threonine phosphatase 4. J. Biol. Chem..

[93] Chen G.I., Tisayakorn S., Jorgensen C., D’Ambrosio L.M., Goudreault M., Gingras A.-C. (2008). PP4R4/KIAA1622 Forms a Novel Stable Cytosolic Complex with Phosphoprotein Phosphatase 4. J. Biol. Chem..

[94] Virshup D.M., Shenolikar S. (2009). From promiscuity to precision: Protein phosphatases get a makeover. Mol. Cell.

[95] Chowdhury D., Xu X., Zhong X., Ahmed F., Zhong J., Liao J., Dykxhoorn D.M., Weinstock D.M., Pfeifer G.P., Lieberman J. (2008). A PP4-phosphatase complex dephosphorylates γ-H2AX generated during DNA replication. Mol. Cell.

[96] Tisayakorn K.W.S. (2009). Structure-Function Analysis of the Trimeric PP4C-PP4R2-PP4R3 Phosphatase Complex. Master’s Thesis.

[97] Seumen C.H., Grimm T.M., Hauck C.R. (2021). Protein phosphatases in TLR signaling. Cell Commun. Signal..

[98] Calvo-Rodriguez M., García-Rodríguez C., Villalobos C., Núñez L. (2020). Role of toll like receptor 4 in Alzheimer’s disease. Front. Immunol..

[99] Hinds Jr T.D., Sánchez E.R. (2008). Protein phosphatase 5. Int. J. Biochem. Cell Biol..

[100] Liu F., Iqbal K., Grundke-Iqbal I., Rossie S., Gong C.-X. (2005). Dephosphorylation of tau by protein phosphatase 5: Impairment in Alzheimer’s disease. J. Biol. Chem..

[101] Gong C.X., Liu F., Wu G., Rossie S., Wegiel J., Li L., Grundke-Iqbal I., Iqbal K. (2004). Dephosphorylation of microtubule-associated protein tau by protein phosphatase 5. J. Neurochem..

[102] Reddy P.H., Beal M.F. (2008). Amyloid beta, mitochondrial dysfunction and synaptic damage: Implications for cognitive decline in aging and Alzheimer’s disease. Trends Mol. Med..

[103] Braithwaite S.P., Stock J.B., Lombroso P.J., Nairn A.C. (2012). Protein phosphatases and Alzheimer’s disease. Prog. Mol. Biol. Transl. Sci..

[104] Zhong J., Liao J., Liu X., Wang P., Liu J., Hou W., Zhu B., Yao L., Wang J., Li J. (2011). Protein phosphatase PP6 is required for homology-directed repair of DNA double-strand breaks. Cell Cycle.

[105] Kajino T., Ren H., Iemura S.-i., Natsume T., Stefansson B., Brautigan D.L., Matsumoto K., Ninomiya-Tsuji J. (2006). Protein phosphatase 6 down-regulates TAK1 kinase activation in the IL-1 signaling pathway. J. Biol. Chem..

[106] Tan P., He L., Cui J., Qian C., Cao X., Lin M., Zhu Q., Li Y., Xing C., Yu X. (2017). Assembly of the WHIP-TRIM14-PPP6C mitochondrial complex promotes RIG-I-mediated antiviral signaling. Mol. Cell.

[107] Li M., Shu H.-B. (2020). Dephosphorylation of cGAS by PPP6C impairs its substrate binding activity and innate antiviral response. Protein Cell.

[108] Stefansson B., Brautigan D.L. (2007). Protein phosphatase PP6 N terminal domain restricts G1 to S phase progression in human cancer cells. Cell Cycle.

[109] Kotak S., Afshar K., Busso C., Gönczy P. (2016). Aurora A kinase regulates proper spindle positioning in *C. elegans* and in human cells. J. Cell Sci..

[110] Mrak R.E., Griffin W.S.T. (2000). Interleukin-1 and the immunogenetics of Alzheimer disease. J. Neuropathol. Exp. Neurol..

[111] Campos A., Clemente-Blanco A. (2020). Cell cycle and DNA repair regulation in the damage response: Protein phosphatases take over the reins. Int. J. Mol. Sci..

[112] Forlenza O.V., de Paula V.J., Machado-Vieira R., Diniz B.S., Gattaz W.F. (2012). Does lithium prevent Alzheimer’s disease?. Drugs Aging.

[113] Chiu C.-T., Chuang D.-M. (2010). Molecular actions and therapeutic potential of lithium in preclinical and clinical studies of CNS disorders. Pharmacol. Ther..

[114] Macdonald A., Briggs K., Poppe M., Higgins A., Velayudhan L., Lovestone S. (2008). A feasibility and tolerability study of lithium in Alzheimer’s disease. Int. J. Geriatr. Psychiatry J. Psychiatry Late Life Allied Sci..

[115] Kamat P.K., Rai S., Nath C. (2013). Okadaic acid induced neurotoxicity: An emerging tool to study Alzheimer’s disease pathology. Neurotoxicology.

[116] Ferrero-Gutiérrez A., Pérez-Gómez A., Novelli A., Fernández-Sánchez M.T. (2008). Inhibition of protein phosphatases impairs the ability of astrocytes to detoxify hydrogen peroxide. Free Radic. Biol. Med..

[117] Puerto Galvis C.E., Vargas Mendez L.Y., Kouznetsov V.V. (2013). Cantharidin-based small molecules as potential therapeutic agents. Chem. Biol. Drug Des..

[118] Sieber M., Baumgrass R. (2009). Novel inhibitors of the calcineurin/NFATc hub-alternatives to CsA and FK506?. Cell Commun. Signal..

[119] Sayas C.L., Ávila J. (2021). GSK-3 and Tau: A key duet in Alzheimer’s disease. Cells.

[120] Venerando A., Ruzzene M., Pinna L.A. (2014). Casein kinase: The triple meaning of a misnomer. Biochem. J..

[121] Borgo C., D’Amore C., Sarno S., Salvi M., Ruzzene M. (2021). Protein kinase CK2: A potential therapeutic target for diverse human diseases. Signal Transduct. Target. Ther..

[122] Dehay B., Bourdenx M., Gorry P., Przedborski S., Vila M., Hunot S., Singleton A., Olanow C.W., Merchant K.M., Bezard E. (2015). Targeting α-synuclein for treatment of Parkinson’s disease: Mechanistic and therapeutic considerations. Lancet Neurol..

[123] Pan J., Zhou L., Zhang C., Xu Q., Sun Y. (2022). Targeting protein phosphatases for the treatment of inflammation-related diseases: From signaling to therapy. Signal Transduct. Target. Ther..

[124] Hayne M., DiAntonio A. (2022). Protein phosphatase 2A restrains DLK signaling to promote proper Drosophila synaptic development and mammalian cortical neuron survival. Neurobiol. Dis..

[125] Gonzalez-Teuber V., Albert-Gasco H., Auyeung V.C., Papa F.R., Mallucci G.R., Hetz C. (2019). Small molecules to improve ER proteostasis in disease. Trends Pharmacol. Sci..

[126] Lemoine L., Leuzy A., Chiotis K., Rodriguez-Vieitez E., Nordberg A. (2018). Tau positron emission tomography imaging in tauopathies: The added hurdle of off-target binding. Alzheimer’s Dement. Diagn. Assess. Dis. Monit..

[127] Seripa D., Solfrizzi V., Imbimbo B.P., Daniele A., Santamato A., Lozupone M., Zuliani G., Greco A., Logroscino G., Panza F. (2016). Tau-directed approaches for the treatment of Alzheimer’s disease: Focus on leuco-methylthioninium. Expert Rev. Neurother..

[128] Cristóbal I., Madoz-Gúrpide J., Manso R., González-Alonso P., Rojo F., García-Foncillas J. (2016). Potential anti-tumor effects of FTY720 associated with PP2A activation: A brief review. Curr. Med. Res. Opin..

[129] Angelopoulou E., Piperi C. (2019). Beneficial effects of fingolimod in Alzheimer’s disease: Molecular mechanisms and therapeutic potential. NeuroMol. Med..

